# Mitomycin C Inhibits Esophageal Fibrosis by Regulating Cell Apoptosis and Autophagy via lncRNA-ATB and miR-200b

**DOI:** 10.3389/fmolb.2021.675757

**Published:** 2021-05-17

**Authors:** Yin Zhang, Qinge Wang, Yuping Xu, Jing Sun, Yanbo Ding, Li Wang, Bingfang Chen, Kewen Sun, Jianping Chen

**Affiliations:** ^1^ Department of Gastroenterology, The Third Affiliated Hospital of Soochow University, Changzhou, China; ^2^ The First People's Hospital of Changzhou, Changzhou, China

**Keywords:** esophageal fibrosis, mitomycin, lncRNA-ATB, MiR-200b, autophagy, apoptosis

## Abstract

Benign esophageal strictures (BESs) frequently results from esophageal fibrosis. The transformation of fibroblasts into fibrocyte is an important cause of fibrosis. The treatment of fibrosis is challenging. Some previous studies have indicated the antifibrotic effect of mitomycin C (MMC). However, the mechanism of action of MMC and its optimal dose for treatment remains unclear. In the present study, the role of MMC in fighting fibrosis and its mechanism was investigated. Human esophageal fibroblast cells (HEFs)were treated without or with MMC, at 2, 5, 10 μg/ml, combining with mimic lncRNA-ATB, miR-200b inhibitor, rapamycin (RAPA), and 3-Methyladenine (3-MA). The cell viability, and cell apoptosis were evaluated. In addition, expression of apoptosis related proteins (caspase8 and caspase3), autophagy related proteins (LC3II and ATG5) and fibrosis related proteins (α-SMA collagen-1 and TGF-β) were also evaluated. Furthermore, autophagosome was observed by transmission electron microscope. Results showed that the expression of lncRNA-ATB was down-regulated and miR-200b was up-regulated after treated with MMC. And MMC induced cell apoptosis and inhibited cell autophagy. On the other hand, RAPA, mimic lncRNA-ATB and miR-200b inhibitor reduced fibrogenic effect of MMC on HEFs. Collectively, this study suggests that MMC inhibited esophageal fibrosis by regulating cell apoptosis and autophagy via downregulating lncRNA-ATB and upregulating miR-200b.

## Introduction

Benign esophageal stricture (BES) is the consequence of esophageal fibrosis, which is caused by a variety of esophageal injuries, such as gastroesophageal reflux, radiotherapy, corrosive substance ingestion, eosinophilic esophagitis and partial esophageal resection (Mizusawa et al., 2019). Recently, esophageal stricture after endoscopic submucosal dissection (ESD) is frequently seen in clinical setting and in standard therapy for early esophageal carcinoma (Ravich, 2017). The stricture formation rate exceeds 90% when the lesions are 75% the size of the circumference (Liao et al., 2018). Esophageal fibrosis results from chronic inflammation, in which inflammatory cells stimulate the fibroblasts, leading to the activation of myofibroblasts and production of extracellular matrix proteins, including collagen (Henderson et al., 2013; Walraven and Hinz, 2018). In addition, myofibroblast, which express smooth-muscle proteins, become contractile. The contraction of these cells contributes to disease pathogenesis and tissue failure. The repair process results in substantial deposition of extracellular matrix components in which normal tissue is replaced with permanent scar tissue (Bülow and Boor, 2019). Hence, the key cellular mediator of fibrosis is fibroblast-myofibroblast transformation and extracellular matrix component disposition.

Mitomycin C (MMC) has been successfully used as an antifibrotic agent to prevent scar formation in glaucoma, lacrimal duct, laryngeal, respiratory, urinary or gastrointestinal tract (Hofheinz et al., 2008; Mizusawa et al., 2019). MMC induced fibroblasts apoptosis and reduced fibrosis by regulating miR-200b and its target gene, RhoE (Sun et al., 2015). Other studies reported that MMC inhibited fibrosis by inducing cell autophagy regulated by certain miRNAs, including miR-200 family (Wang et al., 2017; Hu et al., 2019). Clinical application of MMC on esophageal stricture is used since 2002 (Afzal et al., 2002). Preliminary results were encouraging, however, MMC is an anti-tumor drug that can interfere with natural wound healing and result in serious complications (Wu et al., 2014; Bartel et al., 2016). The mechanism of action of MMC and its optimal dosage for treatment is unknown, that prevent it being regularly used. Therefore, elucidating the mechanism of action and the applicable dosage of MMC is needed.

Based on the encyclopedia of DNA elements initiated in 2003, approximately 65% of genes were transcribed into noncoding RNAs (ncRNAs), and the ncRNAs that possessed more than 200 nucleotides were named long noncoding RNAs (lncRNAs) (Gao et al., 2020). Recently, many studies have shown that lncRNAs are frequently dysregulated in various diseases and have multiple functions in a wide range of biological processes, such as cell proliferation, apoptosis and migration (Yuan et al., 2014). LncRNA-ATB, the lncRNA activated by TGF-β, promotes tumor cell invasion and metastasis, exhibiting oncogenic functions (Xiao et al., 2018). It was reported that the interaction of lncRNA-ATB with miR-200 family promotes the progression of fibrosis in various organs. LncRNA-ATB could act as a fibrogenic cytokine and knockdown of which inhibited the proliferation of keloid fibroblasts by interacting with miR-200c (Zhu et al., 2016). Another study by Fu Na et al. found that lncRNA-ATB promoted liver fibrosis in HCV patients by regulating miR-200a/β-catenin axis (Fu et al., 2017), indicating that lncRNA-ATB could facilitate the progression of fibrosis. However, how MMC interacted with lncRNA-ATB remains controversial.

Recent studies have indicated the protective effects of autophagy on fibrosis in various organs, including renal fibrosis and cardiac fibrosis. Li Huiyan showed that Atg5-mediated autophagy in proximal epithelial cells is a critical host-defense mechanism that prevents renal fibrosis by blocking G2/M arrest (Li et al., 2016). In another study, Trehalose confirmed that Atg5 activates autophagy and improves cardiac remodeling after myocardial infarction. Atg5 increases expression of LC3II, inhibits cardiac fibrosis and increases the ventricular function in mice (Sciarretta et al., 2018).

Given the potential linkage of MMC, lncRNA-ATB, and miR-200b, we explored whether MMC inhibits esophageal fibrosis by regulating lncRNA-ATB, miR-200b and their target genes. In addition, we examined cell autophagy and apoptosis regulated by the expression of lncRNA-ATB and miR-200b. We demonstrated that MMC could induce apoptosis and inhibit autophagy of esophageal fibroblasts by inhibiting lncRNA-ATB, upregulating miR-200b and its target gene LC3 and Atg5, therefore inhibit fibrosis.

## Materials and Methods

### Cells and Cell Culture

The human esophageal fibroblast cells (HEFs) were obtained from iCell Bioscience (iCell Bioscience Inc., Shanghai, China). The HEFs were cultured within Dulbecco's modified Eagle's medium that contained 20% fetal bovine serum (Gibco, United States) and 1% penicillin/streptomycin (Solarbio, China). The cell culture was maintained at the atmosphere with 5% CO_2_ and 95% air, saturated humidity and 37°C. Cells were passaged when the density reached 80–90%. Mimic lncRNA-ATB and miRNA 200b inhibitor were synthesized by the Hanbio Biotechnology. Rapamycin (RAPA, CAS NO.53123-88-9) and 3-Methyladenine (3-MA,CAS NO.5142-23-4) were purchased from Aladdin.

### Cell Viability Assay

The viability of HEFs treated with various concentrations of MMC (2, 5, 10 μg/ml) for 24 or 48 h were evaluated using the cell counting kit-8 assay (Dojindo Laboratories, Japan) according to the manufacturer’s instructions. Cells were treated with PBS in the control group. Cells were plated at a density of 5000 cells/well on a 96-well plate in six replicates. After 24 h, the cells were subjected to various treatments, and then the CCK-8 solution was added to each well and incubated for 2 h at 37°C. Thereafter, the optical density (OD) was measured at 450 nm with a microplate reader (ELx800 Absorbance Microplate Reader, Bio-Tek, United States).

### Flow Cytometry Analysis for Apoptosis

Annexin V/propidium iodide double staining (Invitrogen, United States) was used to detect cell apoptosis. A 6-well plate was used to inoculate HEFs 5*10^5^cells/well. After treatment with 2,5 or 10 μg/ml MMC with or without RAPA (0.4 μmol/ml),3-MA (4 mmol/ml),hs-miRNA-200b (100 nM) and Minic lncRNA-ATB (10 nM), the cells were collected at about 24 h and washed twice with ice-cold PBS. The cells were then resuspended in binding buffer at a concentration of 1 × 10^6^/ml and incubated with annexin V-FITC and propidium iodide for double staining, according to the manufacturer’s instructions. The mixture was incubated in the dark for 15 min at room temperature. Annexin V-FITC was analyzed using the FITC flow cytometry system (Ex = 488 nm; Em = 530 nm) and PI fluorescence intensity was analyzed using PI detection channel (ex = 535 nm; EM = 615 nm). The apoptosis rate in this study represents the total apoptosis rate, including both early and late apoptosis.

### RNA Extraction and Quantitative Real-Time PCR

A 6-well plate was used to inoculate HEFs 5*10^5^ cells/well. After treatment with 2,5 or 10 μg/ml MMC with or without RAPA (0.4 μmol/ml),3-MA (4 mmol/ml),hs-miRNA-200b (100 nM) and Minic lncRNA-ATB (10 nM) for 24 h. The cell plate was added with 600 μl Trizol Reagent (Beyotime, China) and placed on ice for 30 min. RNA was extracted and a Nanodrop 2000 was used to detect the concentration and purity of RNA. The high concentration of RNA was diluted to obtain the final RNA concentration of 200 ng/μl.

Solution containing 2 ug RNA was added with 1 μl oligo (dT)18. Then, make up to 12 μl with deionized water without ribonuclease. The mixture was incubated at 65°C for 5 min, and then quickly cooled on ice. 4 μl of 5 × buffer, 2 μl 10 mm dNTPs, 1 μl RNA inhibitor and 1 μl reverse transcriptase were added in turn, and then the mixture was aspirated. The reverse transcriptase was inactivated at 42°C for 60 min and at 80°C for 5 min. Information of the primers is listed in Table 1.

**TABLE 1:** Primer sequences used in the RT-PCR assay.

Genes	Sequences (5’-3′)
LncRNA-ATB	Forward 5′-CTT​CAC​CAG​CAC​CCA​GAG​A-3′
	Reverse 5′-AAG​ACA​GAA​AAA​CAG​TTC​CGA​GTC-3
GAPDH	Forward 5′-AAAGATGTG CTTCGAGATGTGT-3′
	Reverse 5′-CAC​TTT​GTC​AGT​TAC​CAA​CGT​CA-3′
U6	Forward 5′-CTC​GCT​TCG​GCA​GCA​CA-3′
	Reverse 5′-AAC​GCT​TCA​CGA​ATT​TGC​GT-3′
miR-200b	Forward 5′-CTC​AAC​TGG​TGT​CGT​GGA​GTC​GGC​AAT​TCA​GTT​GAG​TCA​TCA​TT-3′
	Reverse 5′-ACA​CTC​CAG​CTG​GGT​AAT​ACT​GCC​TGG​TAA-3′

The total RNA was reversely transcribed into complementary DNAs (cDNAs) in light of the guidance of PrimeScript RT Reagent Kit (TaKaRa, Japan) under conditions of 37°C for 30 min and 98°C for 5 min. Primer sequences used in the RT-PCR assay were shown in Table S1. 12.5 μl 2× qPCR Mix, 2μl 7.5 μM primer, 2.5μl reverse transcription product, 8.0 μl ddH_2_O, were added in turn. The cDNAs were then amplified via polymerase chain reaction (TaKaRa, Japan). Besides, the reaction conditions for ATB and miR-200b were as follow: (a) degeneration at 95°C for 20 s and (b) 40 cycles of degeneration at 94°C for 5 s, annealing at 55°C for 20 s and extension at 72°C for 20 s. The expressions of lncRNA-ATB and miR-200b were quantified by using 2^−ΔΔCt^ method, with GAPDH and U6 as the internal reference.

### Cell Transfection

For siRNA transfection, Lipofectamine 2000 reagent (Invitrogen, United States) was used according to the manufacturer’s instructions. Cells were trypsinized and seeded in six-well plates at a density of 5*10^5^cells/well for 18 h. Cells that reached 70–90% confluence were transfected with serum-free DMEM medium (iCell Bioscience Inc., China) containing 100 nM siRNA Control, siRNA Beclin 1 for 6 h, followed by recovery in medium containing serum.

### Immunofluorescence

A 12 well plate was used to inoculate HEFs 5*10^5^cells/well. After 70% of confluence, cells were washed with PBS for three times and fixed in 4% paraformaldehyde (Macklin Inc, China) for 30 min. Then, 1 ml 0.2% Triton X 100 (Solarbio lnc, China) was added into the solution for 10 min. The samples were then washed three times with PBS for 10 min each time on the shaker at room temperature. 5% BSA was added for blocking no specific binding for 30 min. Then, the diluted primary antibody (diluted with 1% BSA, Abcam, China) was added and incubated overnight at 4°C. After that, samples were washed three times with PBS, and fluorescent second antibody (1% BSA dilution, Abcam, China) was added and incubated for another 30 min at room temperature. After washing 3 times with PBS, one drop of DAPI was added for 3 min, and another drop of anti-fluorescence quenching agent was added. At last, cells were observed under the fluorescence microscope.

### Western Blot

A six-well plate was taken to inoculate HEF 5*10^5^cells/well. After treatments, cells were washed with PBS for three times. The cells were lyzed with RIPA supplemented with protease inhibitor and protein phosphatase inhibitor. Then, the cell lysate was put on ice for 30 min. The supernatant was centrifuged at 12,000 rpm for 15 min and the protein concentration was detected by Bradford kit. Next, 15 μg/well protein was loaded in 15% SDS-polyacrylamide gel for electrophoresis. Proteins were then transferred onto the polyvinylidene fluoride membrane. After 2 h blocking with 5% bovine serum albumin at room temperature, the diluted primary antibody (Abcam, China) was added for overnight incubation at 4°C. The samples were then washed three times with polysorbate and triethanolamine-buffered saline solution (TBST). Subsequently, the secondary antibody with horseradish peroxidase (1:3000, catalog no.: ab6721, Abcam, China) were added and incubated for another 2 h at room temperature. After rinsing cells with TBST three times, the enhanced chemiluminescence (plusECL) luminescence reagent (Amersham Biosciences) was added for development. Gray value of the protein bands was analyzed by ImageJ software and the data was analyzed by Graphpad.

### Autophagosome Observation by Transmission Electron Microscope

HEFs were inoculated in six-well culture plates and cultured for 24 h. After treatment with different drugs, the cells were fixed with 1% glutaraldehyde and post-fixed with 2% osmium tetroxide. Then, the cell pellets were embedded in Epon resin. Representative areas were chosen for ultrathin sectioning and viewed using a FEI Tecnai G2 Spirit Bio TWIN transmission electron microscope (FEI Co., Netherlands) at an acceleration voltage of 120 kV.

### Statistical Analysis

All the statistical analyses were carried out using the SPSS 19.0 software or GraphPad Prism (7.0). A one-way analysis of variance (ANOVA), followed by a LSD (Least Significant Difference) test were used to compare among different treatment groups. The difference was deemed as statistically significant in case of *P < 0.05, **P < 0.01 and ***P < 0.001.

## Results

### MMCHEFs Induced Cell Apoptosis

**FIGURE 1:**
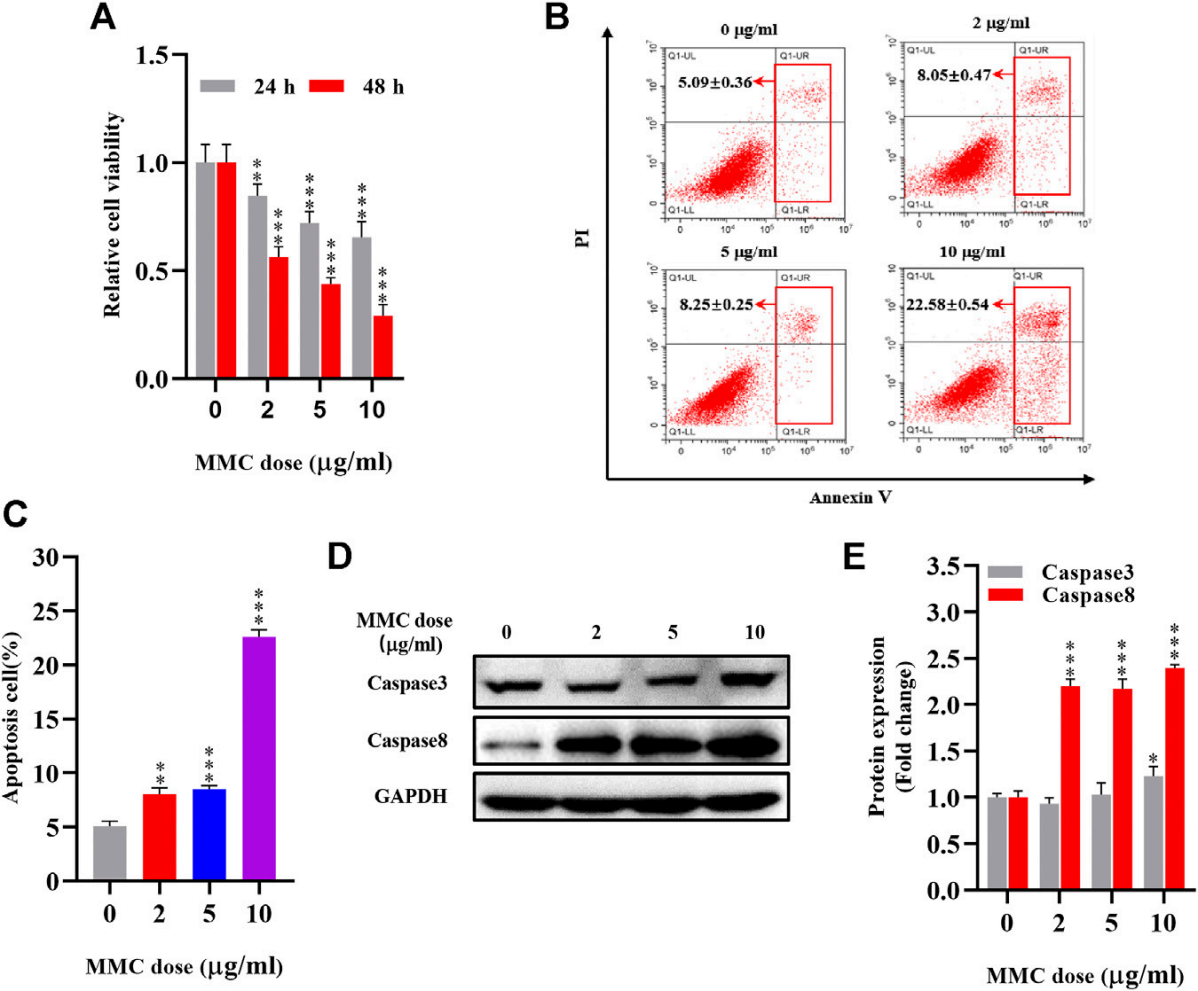
MMC inhibited HEFs proliferation and induced cell apoptosis. HEFs cells were treated with MMC at 2, 5, and 10 μg/ml for 24 or 48 h **(A)** Cell viability was assessed by CCK-8 assay with HEFS treated with or without MMC for 24 or 48 h **(B)** Apoptosis was assessed by annexin V-FITC/PI staining followed by flow cytometry with HEFS treated with or without MMC for 24 h **(C)** Apoptosis positive cells were counted from three independent experiments. **(D)**Western blotting was used to determine the expression levels of Caspase3 and Caspase8 with HEFS treated with or without MMC for 24 h **(E)** Relative protein expression levels by image J statistics from three independent experiments. Data are shown as the means ± SEM from three independent experiments. **P < 0.01,***P < 0.001 vs. Control.

Cells were treated with various concentrations of MMC (0, 2, 5, 10 μg/ml) for 24 or 48 h and evaluated using the CCK-8 assay. As shown in Figure 1A, MMC inhibited the proliferation of these fibroblasts in a time- and dose-dependent manner. A reduction in cell viability was observed 24 h after treatment with MMC at a concentration of 2 μg/ml. After treatment with MMC at 2, 5, 10 μg/ml for 24 h, the cell viability decreased by 15.4%、28.0%、34.7%, respectively (*P* < 0.05). After treatment with MMC at 2, 5, 10 μg/ml for 48 h, it decreased by 43.8%, 56.3%, 71.1%, respectively (*P* < 0.05). Cells treated with the MMC concentration higher than 10 μg/ml led to obvious cell death (result not shown). For the following experiment, the exposure time of MMC was set at 24 h and the highest concentration was 10 μg/ml. Cells treated with 0, 2, 5, 10 μg/ml MMC for 24 h underwent obviously apoptosis, as shown by the cytometric analysis on the annexin V/propidium iodide double staining. Compared with the control group, the percentage of apoptotic cells increased from 5.09% to 8.04%, 8.51%, and 22.57%, respectively, with the red box area (Figures 1B,C).

The proteins related to apoptosis were examined using Western blotting. As showed in Figures 1D,E compared with the control group, MMC treatment with the concentration of 2, 5, 10 μg/ml, resulted in upregulation of Caspase3 by 22.7% after 10 μg/ml MMC treatment (*p* < 0.05). However, Caspase8 was significantly up-regulated by 119.8%, 116.8% and 139.5%, respectively (*p* < 0.05).

### MMC Inhibited HEFs Autophagy and Regulated Expression of lncRNA-ATB and miR-200b

**FIGURE 2:**
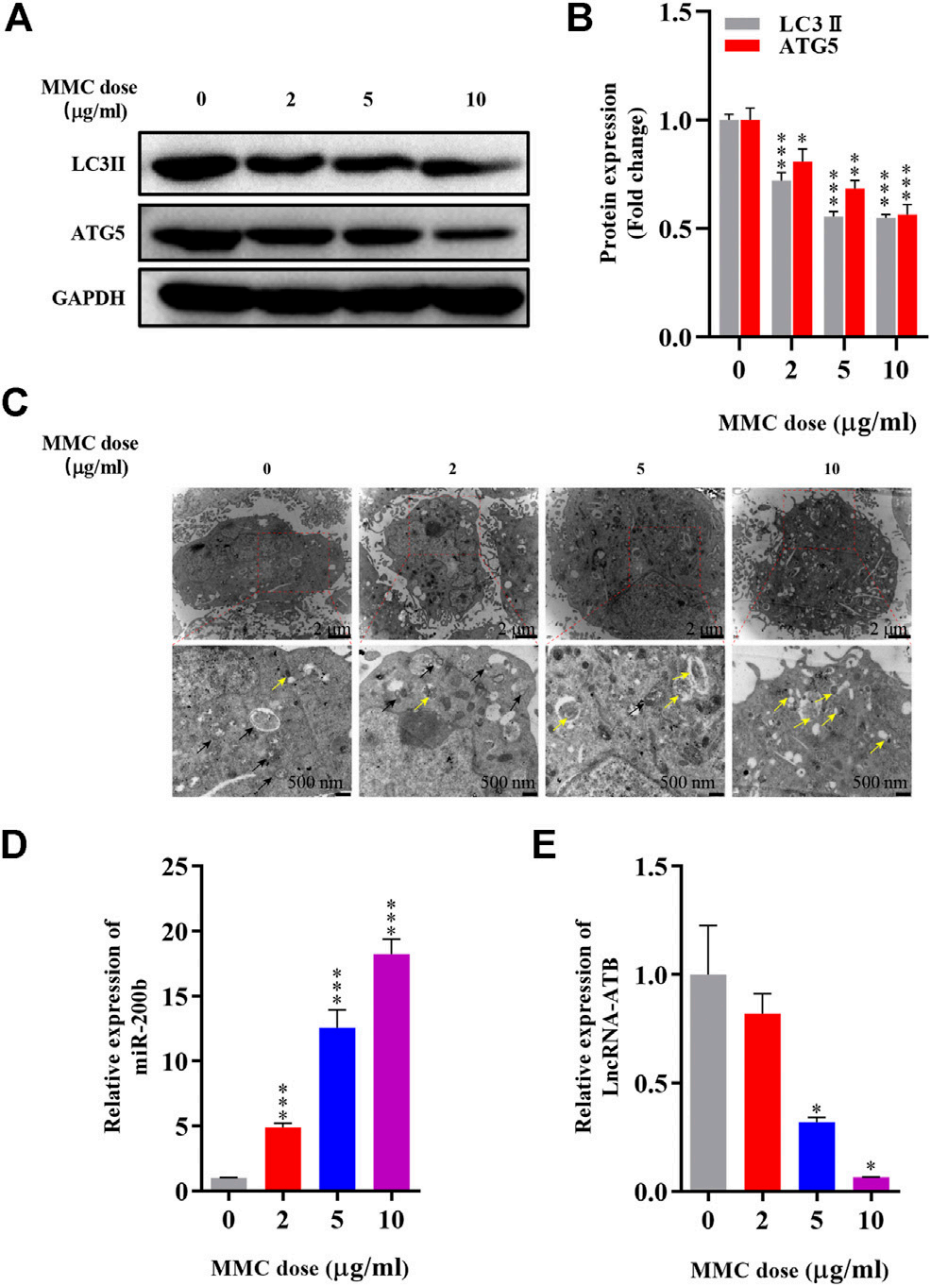
MMC inhibited HEFs autophagy and regulated expression levels of lncRNA-ATB and miR-200b **(A)** Western blotting was used to determine the expression levels of LC3II and ATG5 with HEFS treated with or without MMC for 24 h **(B)** Relative protein expression levels of LC3II and ATG5 compared with the control group. **(C)** Autophagosomes and lysosomes were observed by transmission electron microscope after treatment with MMC at 2, 5, and 10 μg/ml **(D,E)** HEFs cells were treated with MMC at 2, 5, 10 μg/ml, the relative expression of miR-200b and lncRNA-ATB was compared with the control group. Black arrow: Autophagosome; Yellow arrow: lysosome *P < 0.05 **P < 0.01***P < 0.001 vs. Control.

Autophagy related proteins, including LC3 II and ATG5 were significantly down-regulated as shown by Western blot analysis. The expression of LC3 II was 27.9%, 44.6%, 45.2% lower (p< 0.05) and ATG5 was 19.2%, 31.6%, 43.6% lower (*P* < 0.05) compared with the control group (Figures 2A,B). In addition, as shown in Figure 2C, fewer autophagosomes and more lysosomes were observed in MMC treated cells observed by transmission electron microscope after MMC treatment with higher concentration.

Furthermore, to determine the effect of MMC on lncRNA-ATB and miR-200b expression in HEFs, we treated HEFs with 0, 2, 5, or 10 μg/ml MMC for 24 h. The level of lncRNA-ATB and miR-200b was evaluated by qRT-PCR. It was found that the level of lncRNA-ATB in MMC-treated HEFs was significantly downregulated by 17.95%, 68.04% and 93.47%, respectively. In addition, miR-200b was significantly up-regulated by 4.89, 12.56 and 18.23 times, compared with the control group (Figures 2D,E). These results indicated that MMC downregulated lncRNA-ATB expression and upregulated miR-200b expression in HEFs.

### MMC Inhibited the Expression of Fibrotic Marker Proteins in HEFs

**FIGURE 3:**
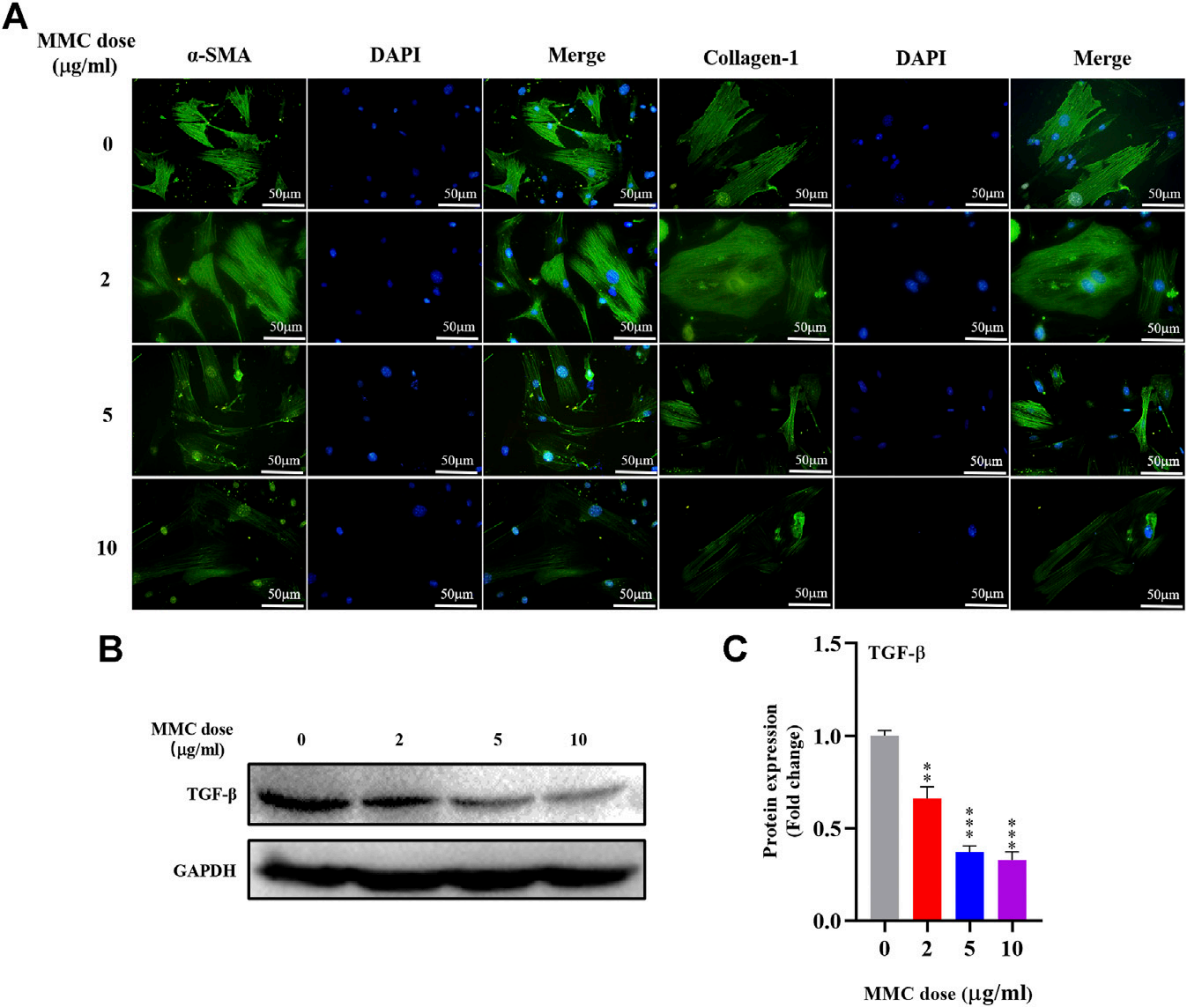
MMC inhibited the expression of fibrotic marker proteins in HEFs. **(A)** Expression of α-SMA and collagen-1 were shown by immunofluorescence after treated with MMC at 2, 5, and 10 μg/ml; **(B)** Western blotting was used to determine the expression levels of TGF-β treated with or without MMC for 24 h. **(C)** Relative protein expression level of TGF-β was shown compared with control group. *P < 0.05 **P < 0.01***P < 0.001.

Immunofluorescence staining was performed on HEFs after treatment with 0, 2, 5 and 10 μg/ml MMC. It was found that the fluorescence intensity of fibrotic marker proteins α-SMA and collagen-1 (green) decreased compared with the control group, indicating that MMC inhibited the process of fibrosis (Figure 3A). And, the fibrogenic related protein, TGF-β, was decreased by 33.9%, 63.0% and 67.2%, after treatment of different concentration of MMC (Figures 3B,C).

### MMC Treatment Combined With Autophagy Agonist or Antagonist, or With lncRNA-ATB Mimic or miR-200b Inhibitor Regulated Cell Apoptosis and Autophagy

**FIGURE 4:**
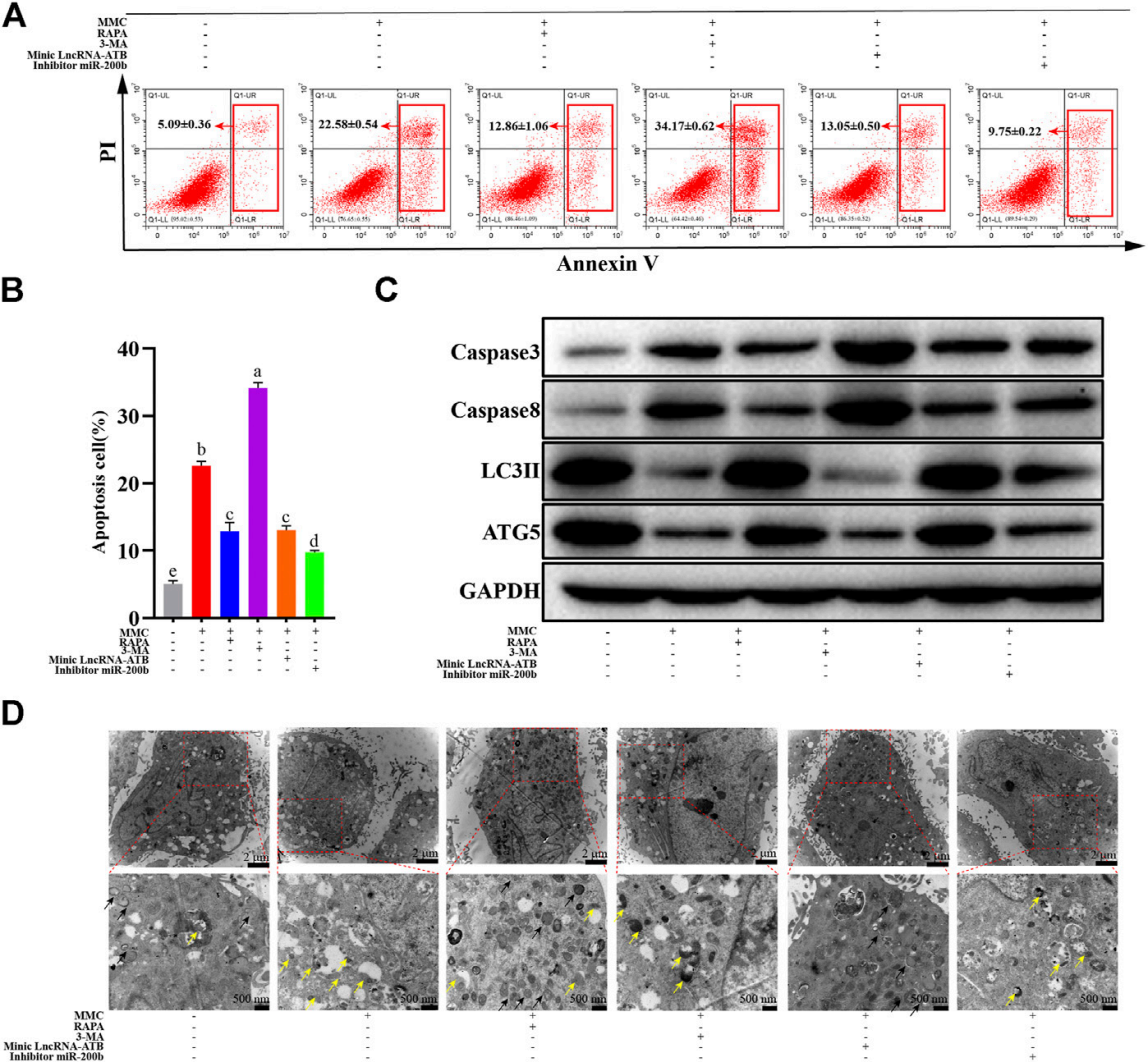
MMC treatment combined with autophagy agonist or antagonist, or lncRNA-ATB mimic or miR-200b inhibitor regulated cell apoptosis and autophagy. **(A)** Apoptosis was assessed by annexin V-FITC/PI staining followed by flow cytometry with HEFS treated with or without MMC for 24 h, MMC + RAPA, MMC+3-MA, MMC + lncRNA-ATB mimic or MMC + miR-200b inhibitor. **(B)** Apoptosis positive cells were counted from the six groups. **(C)** Western blotting was used to determine the expression levels of Caspase3, Caspase8, LC3II and ATG5 after treated with or without MMC for 24 h, MMC + RAPA, MMC+3-MA, MMC + lncRNA-ATB mimic or MMC + miR-200b inhibitor. **(D)** Autophagosomes and lysosomes were observed after treatment with MMC or combined with other drugs. Different letters on the bar chart meant significant difference.

MMC induced cell apoptosis, as shown in Figure 1. MMC treatment combined with autophagy agonist RAPA reduced cell apoptosis in both early and later stage, while the autophagy inhibitor 3-MA promoted cell apoptosis induced by MMC. On the other hand, LncRNA-ATB mimic or miR-200b inhibitor reduced cell apoptosis induced by MMC treatment (Figures 4A,B). These results suggested that MMC induced cell apoptosis can be alleviated by LncRNA-ATB and miR-200b-inducing cell autophagy.

In addition, after treatment with MMC in HEFs, the apoptosis related proteins were upregulated. Meanwhile, the autophagy and fibrosis related proteins were downregulated. Furthermore, combined treatment with MMC and RAPA reduced the expression of caspase3 and caspase8, while increasing the expression of LC3 II and ATG5. In addition, lncRNA-ATB mimic or miR-200b inhibitor had similar effect compared with RAPA, reducing cell apoptosis, and promoting autophagy. On the other hand, 3-MA had the opposite effect on apoptosis, fibrosis and autophagy (Figure 4C).

As shown in Figure 4D, after co-treatment with MMC and RAPA or lncRNA-ATB mimic, the number of autophagosomes increased and lysosomes reduced compared with the MMC group. On the other hand, after co-treatment with MMC and 3-MA or miR-200b inhibitor, no autophagosome was observed and lysosomes also reduced.

### MMC Treatment Combined With Autophagy Agonist or Antagonist, or lncRNA-ATB Mimic or miR-200b Inhibitor Regulated Fibrotic Marker Proteins

**FIGURE 5:**
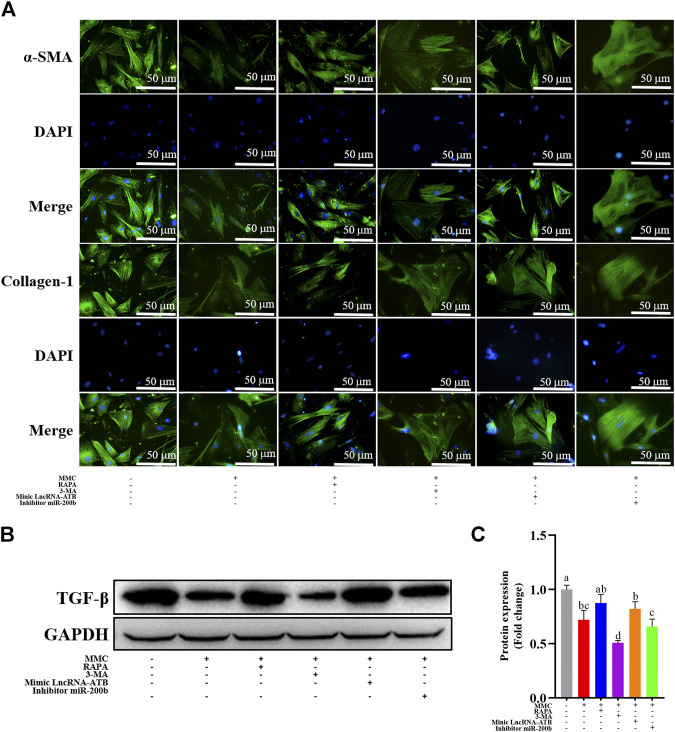
MMC treatment combined with autophagy agonist or antagonist, or lncRNA-ATB mimic or miR-200b inhibitor regulated fibrotic marker proteins. **(A)** Expression of α-SMA and collagen-1 were shown by immunofluorescence after treatment with or without MMC, MMC + RAPA, MMC+3-MA, MMC + lncRNA-ATB mimic or MMC + miR-200b inhibitor. **(B)** Western blotting was used to determine the expression levels of TGF-βtreated with or without MMC, MMC + RAPA, MMC+3-MA, MMC + lncRNA-ATB mimic or MMC + miR-200b inhibitor. **(C)** Relative protein expression level of TGF-β was shown compared with each other. Different letters on the bar chart meant significant difference.

MMC inhibited the expression of fibrotic marker proteins, including TGF-β by Western blotting, α-SMA and Collagen-1 by immunofluorescence analysis. Furthermore, combined treatment with MMC + RAPA, MMC + lncRNA-ATB mimic, MMC + miR-200b inhibitor increased the expression of α-SMA and Collagen-1, compared with MMC group. On the other hand, 3-MA had the opposite effect on the expression of α-SMA and Collagen-1 (Figure 5A). Furthermore, the expression of TGF-β was also up-regulated after treatment with MMC and RAPA, lncRNA-ATB mimic or miR-200b (Figures 5B,C).

## Discussion

Previous studies have demonstrated the antifibrotic effect of MMC after surgery or trauma in various organs. MMC serves as a potent inhibitor of fibroblasts. The antifibrotic mechanisms of MMC on fibroblasts include inhibiting cell proliferation, migration and transformation, and inducing cell apoptosis (Rustagi et al., 2015). The dose of MMC at the concentrations ranging from 0.05–0.4 mg/ml could significantly induce cytotoxicity in intraocular fibroblasts. The concentration of MMC to induce cytotoxicity could be much lower in other organs. A number of studies using MMC in clinic showed its safety and effectiveness (Sui et al., 2017; Chen et al., 2019). However, in an animal study by Wu et al., injection of MMC at the concentrations of 0.5 mg/ml or 0.05 mg/ml was used in porcine models after endoscopic mucosectomy. One pig died from an esophageal perforation after the use of high MMC concentration (Wu et al., 2014). Therefore, optimal concentration of MMC is critical. In this study, MMC inhibited the proliferation of fibroblasts in a time- and dose-dependent manner. Cells treated with MMC at the concentration higher than 10 μg/ml led to cell death. Thus, MMC less than 10 μg/ml for 24 h incubation is the safe concentration for the treatment of fibroblasts.

**FIGURE 6:**
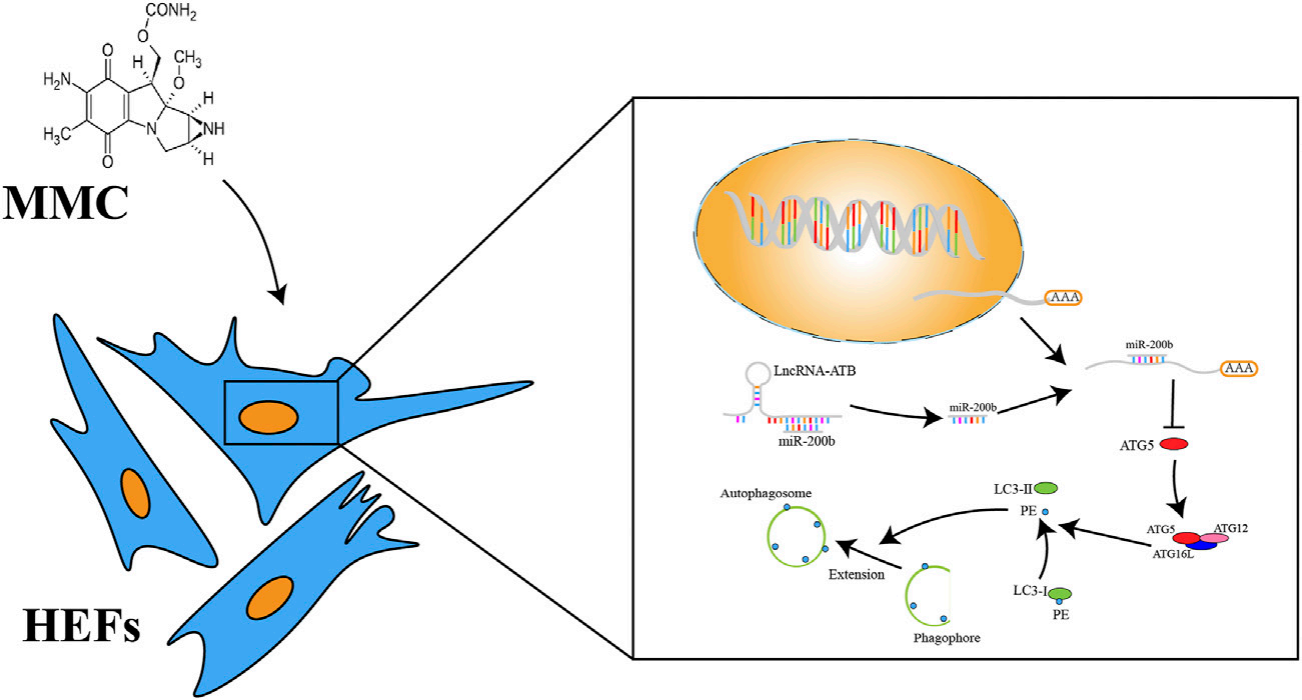
A schema of mechanism of MMC on HEFs. MMC acted on lncRNA-ATB. LncRNA-ATB bound to miR-200b and sequestered miR-200b away from mRNA of ATG5. ATG5 protein led to the downstream change from LC3-I to LC3-II and promoted formation of autophagosome.

In a study published in 2015 by Sun et al., they showed that MMC reduced epidural fibrosis and induced fibroblasts apoptosis by regulating miR-200b and its targeting gene RhoE-2 (9). In another study by Wang et al., they showed that after MMC treatment on scar tissue of laminectomy, the expression of miR-34a, miR-146a and miR-200 were significantly increased, while the levels of miR-16, miR-221 and miR-378a were significantly decreased (10). In addition, the levels of autophagy-related proteins (autophagy protein 5, beclin-1, LC3B-2/1 and p53) were significantly elevated after MMC treatment. These studies suggested that MMC might inhibit fibrosis by regulating autophagy via interacting with certain ncRNAs. Consistently, results in this study also showed that MMC induced cell apoptosis, inhibit autophagy and fibrosis. In this study, we found that MMC could significantly down-regulate the expression of lncRNA-ATB and up-regulate the expression of miR-200b. The expression of apoptosis related proteins (caspase3 and caspase8) was upregulated and expression of fibrosis related proteins (α-SMA and Collagen-1) was downregulated. Furthermore, WB showed the autophagy related proteins (LC3 II and ATG5) were also reduced. These results indicated that MMC could induce fibroblast apoptosis and inhibit cell autophagy and fibrosis. The schematic diagram of mechanism was shown in Figure 6. The results of our study were in consistent with most of the previous studies.

LncRNA-ATB plays vital roles in the progression of various tumors and it is supposed to be an indicator of prognosis for many tumors. For example, in the study by Chuan-Zhuo Wang, lncRNA-ATB promoted autophagy by activating Yes-associated protein and inducing autophagy-related protein 5 in hepatocellular carcinoma (Wang et al., 2019). Some recent studies showed that lncRNA-ATB played a fibrogenic role, and bound to miR-200 family and their downstream target genes (Zhu et al., 2016; Fu et al., 2017). On the other hand, lncRNA-ATB was reported to play its functions by regulating autophagy (Li et al., 2016). In their results by luciferase reporter gene assay, miR-200 family was confirmed as the target of lncRNA-ATB. These results indicated that lncRNA-ATB might drive the process of fibrosis, while miR-200 played an inhibiting role in fibrosis. In the present study, after MMC treatment, the expression of these ncRNAs were dysregulated. We suggested that high expression of lncRNA-ATB and low expression of miR-200b might facilitate the process of esophageal fibrosis.

Some studies also showed that autophagy plays a role on the process of fibrosis. Autophagy is another type of cell death and has been a hotspot in cancer research. Autophagy is related with fibrosis. A study by Zhao et al. suggested that DNMT3A inhibited the expression of miR-200b in cardiac fibroblasts and miR-200b inhibition could activate autophagy and then inhibit the process of fibrosis (Zhao et al., 2018). Another study by Wu et al. showed that autophagy promoted fibrosis and apoptosis in peritoneum fibroblasts in patients with peritoneal dialysis (Wu et al., 2018). So, whether autophagy is a promoter or protector of fibrosis was controversial. In our study, MMC treatment led to inhibition of autophagy, which suggested that fibroblast autophagy promoted the fibrosis process.

We found that LncRNA-ATB mimic and miR-200b inhibitor could partially attenuate the effect of MMC, which could promote autophagy and fibrosis. Meanwhile, the cell apoptosis was significant reduced. After treatment with lncRNA-ATB mimic or miR-200b inhibitor, the expression of apoptosis related proteins (caspase3 and caspase8) was downregulated and expression of fibrosis related proteins (α-SMA, Collagen-1 and TGF-β) was upregulated. Immunofluorescence showed that the expression of α-SMA and collagen-1 reduced. These results also showed that MMC inhibited fibrosis via acting on lncRNA-ATB and miR-200b.

In addition, MMC could inhibit autophagy according to the results from transmission electron microscope and the expression of LC3 II and ATG5. After treatment with MMC, no more autophagosomes were observed by transmission electron microscope. And autophagy related proteins LC3 II and ATG5 were downregulated as shown by WB analysis. However, after treatment with lncRNA-ATB mimic or miR-200b inhibitor, the opposite results were obtained, in which more autophagosomes were observed and expression of LC3 II and ATG5 increased.

The autophagy inducer RAPA and autophagy inhibitor 3-MA were used to evaluate the effect of fibroblast apoptosis and the process of fibrosis. Treatment with RAPA showed similar results with lncRNA-ATB mimic and miR-200b inhibitor. And, 3-MA had the opposite results. These results showed that MMC inhibited fibrosis by regulating cell apoptosis and autophagy via acting on lncRNA-ATB and miR-200b. MMC induced apoptosis and inhibit fibrosis. On the other hand, after co-treatment with RAPA, the cell apoptosis significantly reduced, which also indicated that autophagy might facilitate fibrosis by reducing fibroblast apoptosis.

In summary, this study provides several novel findings on the mechanism by which MMC reduced esophageal fibrosis. First, lncRNA-ATB was significantly downregulated and miR-200b was significantly upregulated in response to MMC treatment. Second, MMC could induce apoptosis and reduce fibrosis by regulating lncRNA-ATB and miR-200b expression. Third, autophagy was important in cell apoptosis and fibrosis. The role of MMC in fibrosis is summarized in Figure 6. Our findings showed that MMC inhibited fibrosis by enhancing cell apoptosis and inhibiting autophagy via upregulating lncRNA-ATB and downregulating miR-200b, suggesting that lncRNA-ATB and miR-200b could be ideal targets for prevention and treatment of esophageal fibrosis and other age-related fibrotic diseases.

## Data Availability

The original contributions presented in the study are included in the article/Supplementary Material, further inquiries can be directed to the corresponding authors.
